# Trajectory of Multiple Chronic Conditions and Associated Factors Among Noninstitutionalized Adults Aged 60 Years or Older in Southern Brazil

**DOI:** 10.5888/pcd21.240082

**Published:** 2024-11-21

**Authors:** Samara Christ Teixeira, Thaynã Ramos Flores, Mariana Otero Xavier, Bruno Pereira Nunes, Elaine Tomasi, Andrea Dâmaso Bertoldi, Flávio Fernando Demarco, Maria Cristina Gonzalez, Renata Moraes Bielemann

**Affiliations:** 1 Postgraduate Program in Food and Nutrition, Federal University of Pelotas, Pelotas, Rio Grande do Sul, Brazil; 2 Postgraduate Program in Epidemiology, Federal University of Pelotas, Pelotas, Rio Grande do Sul, Brazil; 3 Department of Preventive Medicine, Faculty of Medicine, University of São Paulo, São Paulo, Brazil; 4 Department of Nursing in Collective Health, Postgraduate Program in Nursing at the Federal University of Pelotas, Pelotas, Rio Grande do Sul, Brazil

## Abstract

**Introduction:**

The prevalence of chronic conditions is increasing worldwide. The objective of this study was to describe the trajectory of the occurrence of multiple chronic conditions during 6 years of follow-up and investigate their association with demographic, socioeconomic, and behavioral health characteristics of older adults in Southern Brazil.

**Methods:**

We used data from a longitudinal study (the *Como Vai?* study) of noninstitutionalized adults aged 60 or older living in the urban area of Pelotas, Rio Grande do Sul. We assessed the number of chronic conditions based on a list of 24 conditions in 3 interviews, conducted in 2014, 2016–2017, and 2019–2020. We used group-based semiparametric modeling to identify groups of participants based on the number of chronic conditions. For associations with participant characteristics, we performed multinomial logistic regression and considered a low, moderate, and high burden of chronic conditions.

**Results:**

Of the 1,451 older adults in the cohort, 1,098 (75.7%) were included in analysis. Almost one-third (30.9%) had a low burden (2.3 conditions), more than half (52.0%) had a moderate burden (5.6 conditions), and 17.1% had a high burden (9.7 conditions). Men (relative risk [RR] = 6.10; 95% CI, 3.64–10.22), those aged 80 years or older (RR = 2.33; 95% CI, 1.15–4.72), those with no education (RR= 4.78; 95% CI, 2.19–10.45), and former smokers (RR = 1.53; 95% CI, 0.96–2.44) had a higher risk of being classified in the high-burden group than in the low-burden group.

**Conclusion:**

Most older adults belonged to the group with a moderate number of chronic conditions. Several sociodemographic characteristics were associated with belonging to the trajectory with a greater number of conditions.

**Summary**

**What is already known about this topic?**

The prevalence of chronic conditions and multimorbidity is increasing worldwide, particularly among the older population.

**What does this report add?**

We found a high prevalence of chronic conditions in the study sample. Approximately 1 in 5 adults aged 60 years or older lived with approximately 10 simultaneous chronic conditions during the study period, with the greatest risk of these among adults with low levels of education.

**What are the implications for public health practice?**

Because the demand for health services increases as the number of conditions in the population increases, a high prevalence of multiple chronic conditions has substantial effects on the use and cost of health services.

## Introduction

Multimorbidity is the simultaneous presence of 2 or more chronic conditions in the same person (1). It is important to know the prevalence of multimorbidity in the population, because it can influence the population’s quality of life and increase the use and cost of health services (2). Although important, information on multimorbidity, especially in Brazil, is incipient, particularly with regard to understanding trajectories in the development of multiple conditions in the same person (3). Trajectory, in the context of this study, refers to the change in the course of health and illness as a person develops multiple chronic illnesses over time, inherent to the aging process or not.

A systematic review that included 21 studies from 11 high-income countries observed that multimorbidity can affect up to 60% of the world’s older population, reaching 80% among those aged over 80 years (1). In Brazil, according to the 2019 National Health Survey, 47.6% (95% CI, 47.0%–48.3%) of adults, including older adults, reported 1 or more chronic noncommunicable conditions, of whom 12.5% reported 2 conditions, 5.2% reported 3 conditions, and 3.2% reported 4 or more conditions (4). Noncommunicable disease represents the largest disease burden in the world; its prevalence increases with age and socioeconomic vulnerability; that is, older people with worse economic and living conditions tend to have the highest prevalence (4–6).

As multimorbidity increases, so does the need for understanding its causes and associated factors. Most evidence on factors associated with multimorbidity comes from cross-sectional studies that use data from surveys or records of medical visits (1,6–9). The literature has focused on estimating the prevalence of multimorbidity and its determinants, and although some studies have described the occurrence of multimorbidity among older adults, few have verified the trajectories of multimorbidity (10–12).

The objective of this study was to describe the simultaneous occurrence of multiple chronic conditions and multimorbidity trajectories among adults aged 60 years or older in Southern Brazil and investigate their association with demographic, socioeconomic, and behavioral health characteristics during a 6-year period.

## Methods

This study included participants from the Longitudinal Study of Elderly Health, an ongoing cohort study that derives from a cross-sectional survey, *Como Vai?* (Consórcio de Mestrado Orientado para Valorização da Atenção ao Idoso [Master’s Consortium Oriented for the Appreciation of Older Adults Care]), which began in 2014 (13,14). The initial baseline survey had a cross-sectional design and took place from January to August 2014, with the objective of evaluating demographic and economic characteristics and health outcomes among adults aged 60 years or older living in the urban area of the city of Pelotas. Inclusion criteria were adults who were 1) community-dwelling (ie, noninstitutionalized), 2) aged 60 years or older, 3) living in an urban area of Pelotas (~324,000 inhabitants, 93% urban area, ~78,000 adults aged ≥60 y [25%]) (15,16), and 4) physically and mentally able to answer the questionnaire in the absence of a caregiver. The sample size calculation (N = 1,649) was performed to meet all objectives of the baseline study; it accounted for an increase of 15% for possible confounding factors and 10% for losses and refusals. A representative sample was obtained in 2 steps. Initially, 133 census tracts were selected according to size and income. To reach the total estimated sample, 3,745 households in the urban area had to be sampled, with 31 households systematically selected per sector to find at least 12 older adults in each sector (estimate of 0.4 older adults per household).

Interviewers trained to interview and perform anthropometric measurements administered a questionnaire on various aspects of health. Participants were interviewed in their homes, and data were recorded in netbooks. Details on methods used in the *Como Vai*? study can be found elsewhere (13).

A second survey (ie, the first follow-up) of the sample occurred from November 2016 through April 2017. These contacts were conducted by telephone, or for those who could not be contacted by telephone, by home interview. The third survey (ie, the second follow-up) began in September 2019, with only home interviews. These contacts and follow-up were discontinued on March 13, 2020, because of the COVID-19 pandemic and health recommendations for social isolation.

In 2014, at baseline, 1,844 adults aged 60 years or older were located and deemed eligible to participate in the *Como Vai?* study; of these 393 (21.3%) refused to participate or were lost to follow-up, resulting in 1,451 interviewees (78.7%). At first follow-up (2016–2017), 1,161 were interviewed; 153 had been lost to follow-up or refused to participate, and 137 participants died before the interview. At the second follow-up (2019–2020), 537 completed the interview; 8 participants died after the first follow-up interview. We had insufficient time to contact the remaining participants before the study was discontinued due to health recommendations for social distancing due to the COVID-19 pandemic. The analytical sample consisted of 1,098 participants with information from at least 2 survey administrations.

### Dependent variables

The presence of multiple chronic conditions was assessed at baseline and each follow-up through a self-reported medical diagnosis. The survey listed 24 conditions: arthritis, asthma, bronchitis, cancer, constipation, deafness, depression, diabetes, difficulty swallowing, fainting, fecal incontinence, glaucoma, heart failure, high blood pressure, hypercholesterolemia, insomnia, kidney failure, memory problems, osteoporosis, Parkinson disease, rhinitis, seizures, stomach ulcer, or urinary incontinence. The survey asked participants whether (yes or no) they had the condition. The dependent variable, multimorbidity trajectory, was structured according to the number of conditions reported by each participant in the 3 survey administrations.

### Covariates

The independent variables evaluated in 2014 were sex (male, female), age in years (60–69, 70–79, ≥80 y), skin color (white, multiracial), marital status (married/with a partner; without a partner/separated/divorced; widowed), number years of education completed (0, 1–7, ≥8 y), currently working (no, yes), economic class (A/B [high income], C [moderate income], D/E [low income]) (17), leisure-time physical activity (at least 150 minutes per week of moderate or vigorous physical activity was considered active) (18), and smoking (consumption of ≥1 cigarettes per day, every day, in the last 30 days), categorized as never smokers, current smokers, and former smokers. The consumption of alcoholic beverages was assessed through the answer to the yes–no question, “In the last 30 days, have you had any alcoholic drinks?”

### Statistical analysis

In the first stage of analysis, we described the characteristics of the baseline sample and the analytical sample in counts, percentages, and 95% CIs. We then used a group-based semiparametric modeling approach to identify trajectories for multiple occurrences of conditions according to the self-reported presence of the 24 health conditions at the 3 survey points. Group-based trajectory modeling is a specialized form of finite mixture modeling in which clustering of conditions occurs through the use of statistical methods. A polynomial function is used to model the relationship between an attribute (ie, morbidities) and age or time (19–21).

We used Stata’s *traj* procedure (StataCorp LLC) to estimate the model (22). The final analytic sample was defined as participants who had provided information for at least 2 of the 3 survey administrations. We did not exclude participants with missing information from the model because group-based trajectory modeling can handle missing data by using maximum likelihood estimation (20). We considered a zero-inflated Poisson distribution and a censored normal distribution (*cnorm*); *cnorm* provided the better fit for our data. The choice of the number and shape of the trajectories was based not only on the best fit of the model, which was evaluated by using the Bayesian information criterion (BIC), but also on the interpretability of the trajectories obtained (20). Furthermore, selection of the appropriate model was based on the average posterior probability scores for each trajectory group (ie, the probability of a person belonging to each trajectory). According to Nagin, an average probability score should be greater than 0.70 for all groups (20).

In the second stage of analysis, we used analysis of variance tests (continuous variables) and χ^2^ tests (categorical values) to compare the characteristics of trajectory groups. We estimated effect measures by using multinomial logistic regression, which generated relative risks (RRs) and 95% CIs. We defined the group with a low burden of conditions as the reference group. The statistical adjustment was based on a hierarchical model constructed in 2 levels. Demographic and socioeconomic variables comprised the first level; variables with *P* values ≤.20 remained in the adjustment model. Behavioral variables were added in the second level along with characteristics that remained from the first level. Variables with *P* values ≤.20 were kept in the final model, controlling for potential confounders at the same level and at the level above. To avoid excluding important confounders, the significance level to admit significant associations was set at ≤.05. We applied the likelihood ratio test to test whether the independent variable was significant for the model (Testparm *P* value ≤ .05). We performed all statistical analyses in Stata version 13.0 (StataCorp LLC).

### Attrition analysis

We conducted an attrition analysis to compare multimorbidity trajectories among participants who provided information on comorbidities at all 3 study administration points (n = 440) to assess the robustness of the results.

The first step was to model multimorbidity trajectories by specifying 2-, 3-, 4-, and 5-group models. Moving from the 3-group to the 4-group model did not improve the modeling (BIC values were similar). The 4- and 5-group models produced 1 or more groups with a very small (<10%) proportion of the observations.

Given the high rate of attrition in the second follow-up survey (2019–2020) resulting from the COVID-19 pandemic, we also sought to compare the characteristics of participants in the second follow-up survey with the characteristics of participants in the first follow-up survey (2016–2017) regarding multimorbidity.

### Ethics statement

All stages of the study were approved by the Research Ethics Committee of the Faculty of Medicine of the Universidade Federal de Pelotas (CAAE: 54141716.0.0000.5317). The research participants, or their guardians, signed the free and informed consent form, which guarantees confidentiality of data. For participants interviewed via telephone (2016–2017), acceptance to answer the questionnaire was provided by verbal assent via telephone.

## Results

At baseline, most respondents were female (63.0%), were aged 60 to 69 years (52.3%), reported white skin color (83.7%), had a partner (52.7%), had 1 to 7 years of education (54.4%), belonged to economic class C (52.5%), and did not work (80.4%) (Table 1). At baseline, 75.2% indicated being sedentary, 54.0% had never smoked, and 78.8% reported not drinking alcohol. We found lower participation rates in the analytical sample than in the baseline sample for the following variables: sex, age, skin color, marital status, educational background, economic class, work status, physical activity, smoking status, and alcohol consumption.

**Table 1:** Demographic, Socioeconomic, and Behavioral Health Characteristics of Participants in the *Como Vai*? Study at Baseline and in the Analytical Sample, Pelotas, Brazil^a^

Variable	No. (%) [95% CI]	
	Baseline^b^ (n = 1,451)	Analytical Sample^c^ (n = 1,098)
**Demographic and socioeconomic**		
**Sex**		
Female	914 (63.0) [60.5–65.4]	703 (64.0) [61.1–66.8]
Male	537 (37.0) [34.5–39.5]	395 (36.0) [33.2–38.9]
**Age group, y**		
60–69	756 (52.3) [49.7–54.8]	612 (55.8) [52.9–58.8]
70–79	460 (31.8) [29.5–34.3]	347 (31.7) [29.0–34.5]
≥80	230 (15.9) [14.1–17.9]	137 (12.5) [10.7–14.6]
**Skin color^d^**		
White	1,211(83.7) [81.7–85.5]	916 (83.5) [81.2–85.6]
Multiracial	236 (16.3) [14.5–18.3]	181 (16.5) [14.4–18.8]
**Marital status**		
Married/with a partner	763 (52.7) [50.1–55.3]	613 (55.9) [52.9–58.8]
Without a partner/separated/divorced	225 (15.6) [13.8–17.5]	171 (15.6) [13.5–17.9]
Widow(er)	459 (31.7) [29.3–34.2]	313 (28.5) [25.9–31.3]
**No. of years of education completed**		
0	196 (13.6) [11.9–15.5]	140 (12.8) [11.0–15.0]
1–7	782 (54.4) [51.8–57.0]	579 (53.1) [50.1–56.1]
≥8	459 (32.0) [29.6–34.4]	371 (34.0) [31.3–36.9]
**Economic class^e^**		
A/B	483 (35.2) [32.2–37.8]	375 (36.1) [33.2–39.1]
C	720 (52.5) [49.8–55.1]	554 (53.4) [50.3–56.4]
D/E	169 (12.3) [10.8–14.2]	109 (10.5) [8.8–12.5]
**Currently working**		
Yes	264 (19.6) [17.5–21.8]	224 (21.7) [19.3–24.3]
No	1,084 (80.4) [78.2–82.4]	807 (78.3) [75.6–80.7]
**Behavioral**		
**Leisure-time physical activity**		
Sedentary (<150 minutes moderate or vigorous physical activity per week)	1,047 (75.2) [72.9–77.4]	786 (73.2) [70.4–75.7]
Active (≥150 minutes moderate or vigorous physical activity per week)	345 (24.8) [22.6–27.1]	288 (26.8) [24.2–29.5]
**Smoking status^f^**		
Never smoker	781 (54.0) [51.4–56.6]	620 (56.5) [53.6–59.4]
Current smoker	182 (12.6) [11.0–14.4]	130 (11.9) [10.1–13.9]
Former smoker	483 (33.4) [31.0–36.0]	347 (31.6) [28.9–34.4]
**Alcohol consumption**		
No	1,138 (78.8) [76.6–80.8]	829 (75.6) [73.0–78.1]
Yes	307 (21.2) [19.2–23.4]	267 (24.4) [21.9–27.0]

a Data source: Longitudinal Study of Elderly Health — *Como Vai?* study (13). The survey listed 24 conditions: arthritis, asthma, bronchitis, cancer, constipation, deafness, depression, diabetes, difficulty swallowing, fainting, fecal incontinence, glaucoma, heart failure, high blood pressure, hypercholesterolemia, insomnia, kidney failure, memory problems, osteoporosis, Parkinson disease, rhinitis, seizures, stomach ulcer, or urinary incontinence.

b Baseline survey was administered from January through August 2014.

c Participants who reported data on multiple chronic conditions at ≥2 study administration points, including baseline (2014), first follow-up (November 2016 through April 2017), and second follow-up (September 2019 through March 13, 2020). Some categories do not add up to 1,098 because data are missing (eg, participant decided to not to answer question, participant did not know how to answer question).

d Choices were White, Black, Brown, Yellow, Indigenous; Black, Brown, Yellow, and Indigenous were combined into multiracial.

e A/B, high income; C, moderate income; D/E, low income, per Associação Brasileira de Empresas de Pesquisa (17).

f Smoking defined as consumption of ≥1 cigarettes per day, every day, in the last 30 days.

### Identification of trajectories

The model with 3 trajectories emerged as the most appropriate and parsimonious model based on the parameters and the interpretability of the trajectories obtained. Group 1 (low burden of chronic conditions; average of approximately 2.3 chronic conditions; n = 339) comprised 30.9% of the sample, group 2 (moderate burden of chronic conditions; average of approximately 5.6 chronic conditions; n = 571) comprised more than half of the sample (52.0%), and group 3 (high burden of chronic conditions; average of 9.7 chronic conditions; n = 188) comprised 17.1% of the sample (Figure ).

**Figure:**
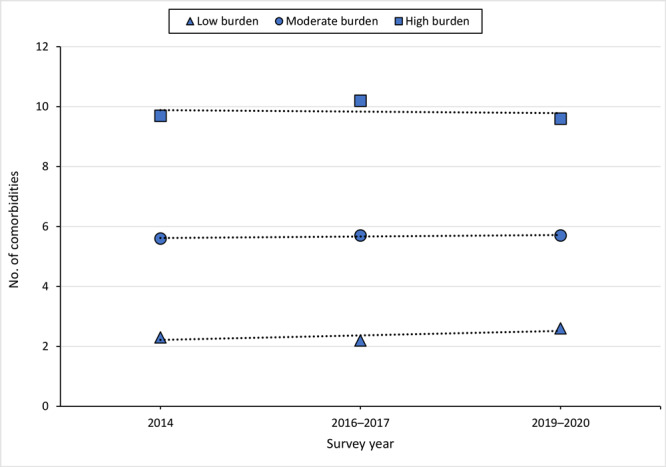
Trajectories of chronic conditions according to the number of conditions among participants in the *Como Vai*? study, Pelotas, Brazil. Of 1,098 study participants, 339 (30.9%) were categorized as having a low burden of chronic conditions, 571 (52.0%) moderate, and 188 (17.1%) high.

**Table:** 

Level	Mean (range) no. of chronic conditions, by year		
	2014	2016	2019
Low (n = 339)	2.3 (0–6)	2.2 (0–6)	2.6 (0–7)
Moderate (n = 571)	5.6 (0–11)	5.7 (1–10)	5.7 (1–13)
High (n = 188)	9.7 (3–16)	10.2 (5–16)	9.6 (5–17)

At baseline, the average number of chronic conditions reported per study participant was 5.0. The number of chronic conditions reported by participants increased at follow-up. At baseline and at first follow-up, the number of conditions in group 1 ranged from 0 to 6 conditions; at second follow-up, the maximum value increased to 7 conditions. In group 2, the number of conditions ranged from 0 to 11 at baseline, 1 to 12 at first follow-up, and 1 to 13 at second follow-up. In group 3, the number of conditions ranged from 3 to 16 at baseline, 5 to 16 at first follow-up, and 5 to 17 at second follow-up.

### Factors associated with trajectories of multiple chronic conditions

In the high-burden group, participants were more likely to be men than women (22.6% vs 7.3%), be aged 80 years or more than aged 70 to 79 years or 60 to 69 years (24.1% vs 18.7% or 14.5%, respectively), identify as multiracial (19.9% vs 16.5% white), be widows or widowers than married or without a partner/separated/divorced (23.6% vs 15.8% or 14.0%, respectively), have no education than have 1 to 7 years or 8 years or more (25.7% vs 20.6% or 7.8%, respectively), be in economic class D/E than in C or A/B (21.1% vs 19.3% or 12.3%, respectively), be not currently working than working (19.2% vs 8.9%), be sedentary than active (19.1% vs 12.2%), be a never smoker than current smoker or former smoker (18.7% vs 12.3% or 15.8%, respectively), and not consume alcohol than consume alcohol (19.8% vs 8.6%) (Table 2).

**Table 2:** Multimorbidity Trajectory Group, by Demographic, Socioeconomic, and Behavioral Health Characteristics of Participants at Follow-Up (N = 1,098) in the *Como Vai*? Study, Pelotas, Brazil^a^

Characteristic	Trajectory group^b^		
	Low burden	Moderate burden	High burden
**All participants, no (%)**	339 (30.9)	571 (52.0)	188 (17.1)
**Demographic and socioeconomic**			
**Sex**			
Female	43.8 (39.0–48.8)	48.9 (43.9–53.8)	7.3 (5.1–10.4)
Male	23.6 (21.0–27.0)	53.8 (50.1–57.4)	22.6 (19.7–25.9)
**Age group, y**			
60–69	36.3 (32.5–40.2)	49.2 (45.2–53.2)	14.5 (12.6–17.6)
70–79	26.8 (22.4–31.7)	54.5 (49.2–60.0)	18.7 (15.0–23.2)
≥80	16.8 (11.4–24.1)	59.1 (50.6–67.1)	24.1 (17.6–32.1)
**Skin color^c^**			
White	30.3 (27.5–33.4)	53.2 (50.0–56.4)	16.5 (14.2–19.0)
Multiracial	33.7 (27.1–41.6)	46.4 (39.2–54.0)	19.9 (14.7–26.4)
**Marital status**			
Married	35.1 (31.4–39.0)	50.9 (47.0–54.9)	14.0 (11.5–17.0)
Without a partner/separated/divorced	34.5 (28.0–42.0)	49.7 (42.2–57.2)	15.8 (11.0–22.1)
Widowed	20.8 (16.6–26.0)	55.6 (50.0–61.0)	23.6 (19.2–28.7)
**No. of years of education**			
0	23.6 (17.2–31.4)	50.7 (42.4–59.0)	25.7 (19.1–33.7)
1–7	28.8 (25.3–32.7)	50.6 (46.5–54.7)	20.6 (17.4–24.1)
≥8	36.7 (31.9–41.7)	55.5 (50.4–60.5)	7.8 (5.5–11.0)
**Economic class^d^**			
A/B	34.4 (29.7–39.4)	53.3 (48.2–58.4)	12.3 (9.3–16.0)
C	29.4 (26.0–33.3)	51.3 (47.1–55.4)	19.3 (16.2–22.8)
D/E	25.7 (18.3–35.5)	53.2 (43.7–62.5)	21.1 (14.4–29.9)
**Currently working**			
Yes	41.5 (35.2–48.1)	49.6 (43.0–56.1)	8.9 (5.8–13.5)
No	28.3 (25.2–31.5)	52.5 (49.1–56.0)	19.2 (16.6–22.1)
**Behavioral health**			
**Physical activity**			
Sedentary (<150 minutes moderate or vigorous physical activity per week)	29.6 (26.5–32.9)	51.3 (47.8–54.8)	19.1 (16.5–22.1)
Active (≥150 minutes moderate or vigorous physical activity per week)	34.0 (28.8–39.7)	53.8 (48.0–60.0)	12.2 (8.8–16.5)
**Smoking**			
Never smoker	28.9 (25.4–33.0)	52.4 (48.5–56.3)	18.7 (15.8–22.0)
Current smoker^e^	43.1 (35.0–52.0)	44.6 (36.2–53.4)	12.3 (7.6–19.3)
Former smoker	30.0 (25.4–35.0)	54.2 (48.9–59.4)	15.8 (12.4–20.1)
**Alcohol consumption**			
No	29.3 (26.3–32.5)	50.9 (47.5–54.3)	19.8 (17.2–22.6)
Yes	36.0 (30.4–41.2)	55.4 (49.4–61.3)	8.6 (6.0–12.7)

a Data source: Longitudinal Study of Elderly Health — *Como Vai?* study (13). The survey listed 24 conditions: arthritis, asthma, bronchitis, cancer, constipation, deafness, depression, diabetes, difficulty swallowing, fainting, fecal incontinence, glaucoma, heart failure, high blood pressure, hypercholesterolemia, insomnia, kidney failure, memory problems, osteoporosis, Parkinson disease, rhinitis, seizures, stomach ulcer, or urinary incontinence.

b Groups: low burden of disease (~2.3 conditions), moderate burden (~5.6 conditions), high burden (~9.7 conditions). All values are % (95% CI) unless otherwise indicated.

c Choices were White, Black, Brown, Yellow, Indigenous; Black, Brown, Yellow, and Indigenous were combined into multiracial.

d A/B, high income; C, moderate income; D/E, low income, per Associação Brasileira de Empresas de Pesquisa (17).

e Smoking defined as consumption of ≥1 cigarettes per day, every day, in the last 30 days.

Men were twice as likely as women to have a moderate burden (RR = 2.00; 95% CI, 1.44–2.77) and 6.1 (95% CI, 3.64–10.22) times as likely to have a high burden of chronic conditions (Table 3). Participants aged 80 years or older, compared with participants aged 60 to 69 years, were 2.52 (95% CI, 1.44–4.39) times as likely to have a moderate burden and 2.33 (95% CI, 1.15–4.72) times as likely to have a high burden of chronic conditions. Participants with no education had a 25% higher risk (RR = 1.25; 95% CI, 0.70–2.25) of having a moderate burden than participants with 8 or more years of education, but 95% CIs indicate that the difference between these 2 groups was not significant. Additionally, participants with no education were 4.78 (95% CI, 2.19–10.45) times as likely as participants with 8 or more years of education to have a high burden. Former smokers, compared with never smokers, had a 37% higher risk (RR = 1.37; 95% CI, 0.98–1.92) of having a moderate burden and a 53% higher risk of having a high burden (RR = 1.53; 95% CI, 0.96–2.44) (Table 3). 

**Table 3:** Unadjusted and Adjusted Analyses of Multimorbidity Trajectories, by Demographic, Socioeconomic, and Behavioral Health Characteristics of Participants (N = 1,098) in the *Como Vai*? Study, Pelotas, Brazil^a^

Variables	Moderate burden of chronic conditions^b^				High burden of chronic conditions^b^			
	Unadjusted, RR (95% CI)	*P* value^c^	Adjusted, RR (95% CI)	*P* value^c^	Unadjusted, RR (95%CI)	*P* value^c^	Adjusted, RR (95%CI)	*P* value^c^
**First level^d^**								
**Sex**								
Female	1 [Reference]	<.001	1 [Reference]	<.001	1 [Reference]	<.001	1 [Reference]	<.001
Male	2.01 (1.49–2.72)		2.00 (1.44–2.77)		5.70 (3.51–9.27)		6.10 (3.64–10.22)	
**Age group, y**								
60–69	1 [Reference]	<.001	1 [Reference]	.02^e^	1 [Reference]	.02	1 [Reference]	.02^e^
70–79	1.50 (1.11–2.03)		1.37 (0.97–1.93)		1.74 (1.17–2.60)		1.41 (0.88–2.25)	
≥80	2.60 (1.58–4.26)		2.52 (1.44–4.39)		3.58 (2.00–6.43)		2.33 (1.15–4.72)	
**Skin color^f^**								
White	1 [Reference]	.24	1 [Reference]	.21	1 [Reference]	.34	1 [Reference]	.33
Multiracial	1.27 (0.86–1.88)		1.28 (0.86–1.90)		1.29 (0.77–2.19)		1.30 (0.77–2.19)	
**Marital status**								
Married	1 [Reference]	.21	1 [Reference]	.80^e^	1 [Reference]	.37	1 [Reference]	.80^e^
Without a partner/separated/divorced	1.01 (0.69–1.47)		1.18 (0.77–1.81)		0.87 (0.52–1.47)		1.14 (0.63–2.07)	
Widow(er)	1.86 (1.20–2.88)		1.38 (0.84–2.26)		2.49 (1.41–4.37)		1.30 (0.68–2.47)	
**No. of years of education completed**								
0	1.42 (0.89–2.26)	.44	1.25 (0.70–2.25)	<.001	5.12 (2.75–9.51)	<.001	4.78 (2.19–10.45)	<.001
1-7	1.16 (0.87–1.55)		1.08 (0.75–1.57)		3.34 (2.10–5.32)		3.54 (2.00–6.29)	
≥8	1 [Reference]		1 [Reference]		1 [Reference]		1 [Reference]	
**Economic class^g^**								
A/B	1 [Reference]	.97	1 [Reference]	.98^e^	1 [Reference]	.87	1 [Reference]	.98^e^
C	1.12 (0.84–1.51)		0.99 (0.70–1.42)		1.84 (1.21–2.79)		0.87 (0.52–1.46)	
D/E	1.34 (0.81–2.21)		1.04 (0.58–1.89)		2.30 (1.21–4.40)		0.84 (0.39–1.83)	
**Currently working**								
Yes	1 [Reference]	.51	1 [Reference]	.25^e^	1 [Reference]	.08	1 [Reference]	.25^e^
No	1.13 (0.79–1.60)		1.14 (0.80–1.63)		1.66 (0.94–2.92)		1.62 (0.91–2.88)	
**Second level^h^**								
**Physical activity**								
Sedentary (<150 minutes moderate or vigorous physical activity per week)	1 [Reference]	.71	1 [Reference]	.58	1 [Reference]	.59	1 [Reference]	.72
Active (≥150 minutes moderate or vigorous physical activity per week)	0.94 (0.69–1.29)		0.92 (0.67–1.26)		1.14 (0.71–1.82)		1.09 (0.68–1.75)	
**Smoking**								
Never smoker	1 [Reference]	.10	1 [Reference]	.02^e^	1 [Reference]	.10	1 [Reference]	.02^e^
Current smoker^i^	0.57 (0.38–0.86)		0.67 (0.43–1.03)		0.44 (0.24–0.81)		0.60 (0.31–1.14)	
Former smoker	1.00 (0.74–1.35)		1.37 (0.98–1.92)		0.82 (0.55–1.22)		1.53 (0.96–2.44)	
**Alcohol consumption**								
No	1 [Reference]	.38	1 [Reference]	.08^e^	1 [Reference]	.11	1 [Reference]	.08^e^
Yes	1.16 (0.83–1.60)		1.17 (0.84–1.63)		0.64 (0.38–1.10)		0.67 (0.39–1.14)	

Abbreviation: RR, risk ratio.

a Data source: Longitudinal Study of Elderly Health — *Como Vai?* study (13). The survey listed 24 conditions: arthritis, asthma, bronchitis, cancer, constipation, deafness, depression, diabetes, difficulty swallowing, fainting, fecal incontinence, glaucoma, heart failure, high blood pressure, hypercholesterolemia, insomnia, kidney failure, memory problems, osteoporosis, Parkinson disease, rhinitis, seizures, stomach ulcer, or urinary incontinence.

b Analyses used low burden of disease (~2.3 conditions) as a reference group for moderate burden (~5.6 conditions) and high burden (~9.7 conditions).

c Determined by Wald χ^2^ test.

d First level: Distal level in the hierarchical statistical adjustment model to test for possible confounding factors and statistical interaction of the independent variables with variance.

e *P* value determined by testparm, a statistical test that verifies the significance of the exposure variable considering the others included in the statistical regression model.

f Choices were White, Black, Brown, Yellow, Indigenous; Black, Brown, Yellow, and Indigenous were combined into multiracial.

g A/B, high income; C, moderate income; D/E, low income, per Associação Brasileira de Empresas de Pesquisa (17).

h Second level: Proximal level in the hierarchical statistical adjustment model to test for possible confounding factors and statistical interaction of the independent variables with variance.

i Smoking defined as consumption of ≥1 cigarettes per day, every day, in the last 30 days.

### Attrition analysis

In the attrition analysis, 440 participants had data on chronic conditions in all 3 surveys. In this subsample, as in the primary analysis, we identified 3 trajectories (Appendix), and the proportion of participants in each group was similar to the proportion in the primary analysis: 117 (26.6%) had a low burden, 254 (57.7%) had a moderate burden, and 69 (15.7%) had a high burden.

According to demographic, socioeconomic, and behavioral health characteristics, the proportions of participants in the second follow-up survey were similar to the proportions in the baseline and first follow-up surveys.

## Discussion

This study compared the trajectories of multimorbidity and associated factors in a cohort of adults aged 60 or older during a 6-year period. We identified 3 groups of multimorbidity trajectories. Approximately one-third of study participants had an average of 2.3 conditions during the study period, more than one-half had an average of 5.6 conditions, and 17% had an average of approximately 9.7 conditions simultaneously.

These results reflect a population with a high prevalence of multiple chronic conditions. This high prevalence has direct economic effects on individuals and the health care system, since the demand for health services increases as the number of conditions in the population increases (23,24). A study in Switzerland on multimorbidity, health care use, and health care costs in an older population showed a 32.6% increase in outpatient and hospital costs for each additional chronic condition. The study also showed significant interactions between age and sex: total health care costs increased by 9% to 10% in men aged 70 and 84, respectively, when compared with men aged 65 and 69 years (23).

The 3 multimorbidity groups found in our study differed according to demographic, socioeconomic, and behavioral health characteristics. The high-burden group had a higher percentage of men (vs women), participants aged 80 years or older (vs 60–79 y), participants with no education (vs those with ≥1 y), and former smokers. We observed greater heterogeneity in the associations for the risk of classification in the low-burden group and moderate-burden group.

Our results shed some light on the influences of social stratification in older age. For example, sociodemographic factors such as sex, age, education, and behavioral factors such as physical activity, smoking, and alcohol consumption were related to a worse trajectory of multimorbidity. The direct relationship between socioeconomic indicators and multimorbidity denotes its relevance in the occurrence and maintenance of social inequities (24,25) and increases the challenges of the health system in managing multimorbidity in the older population (26). In general, the health care system is configured for single conditions rather than multiple conditions (24,26), especially for the older population.

Our findings contradict what has been reported by other studies — that the occurrence of multiple conditions is higher among women than men (1,8,27,28). Compared with men, women usually receive more health guidance, demonstrate higher levels of self-care, and have healthier habits throughout their lives (29).

Another important finding is that an increasing number of older adults are living with multiple conditions simultaneously. In our study, age was significantly associated with multimorbidity trajectories; in the high-burden group, the largest percentage of participants were those aged 80 years or older. As longevity increases, more chronic conditions emerge, and the decline in death rates due to better management and treatment of chronic conditions results in more people surviving for a longer time (30).

Our study demonstrated that a higher level of education contributed to a lower risk of classification in the high-burden group. According to the National Research Council and the Institute of Medicine, education is one of the most important social determinants of health (31), and it has direct effects on health-related factors (32). A study on the association of multimorbidity and education in Germany found that educational level was an important risk factor for the occurrence of multiple diseases; compared with the highest category of educational attainment, the lowest category was significantly associated with an increased probability of multimorbidity in men and women (33). Our study found greater levels of multimorbidity among participants with less education, in line with previous studies (1,8).

Contrary to what we expected, economic class was not significantly associated with multimorbidity trajectories, whereas a low level of education was. Access to formal education, which provides greater access to information, was shown to be more important than access to goods to not be classified in the high-burden group. Education can bring autonomy in care that is not related to economic power. Results on the association of multimorbidity and income have been controversial, however. A study that compared the prevalence of multimorbidity in low-income and middle-income countries showed a positive but nonlinear relationship between the countries’ gross domestic product and the prevalence of multimorbidity (7). Other studies observed a higher risk of multimorbidity among low-income individuals (8,34). Furthermore, a longitudinal study showed that multimorbidity trajectories differed by income level, with a more pronounced trajectory among those with lower income than among those with higher income (35).

Our study corroborates the results of studies showing that smoking and a sedentary lifestyle are determinants of multimorbidity (8,28). We showed that former smokers were at greater risk than never smokers of being classified in the moderate-burden group or high-burden group. Smoking cessation may have occurred after the diagnosis of several comorbidities or smoking cessation may be associated with a higher survival rate, since cessation increases longevity, even if people live with several chronic diseases after cessation. Likewise, a lower risk among current smokers than former smokers of belonging to a higher disease trajectory may be associated with early mortality, since a greater proportion of smoking participants may have died during follow-up. This association was demonstrated in a previous study. In that study, which used data collected through 2017 from the *Como Vai?* sample, almost 10% (n = 145) of participants died during an average of 2.5 years of follow-up, with current smoking (RR = 2.1; 95% CI, 1.2–3.8) one of the behaviors associated with the highest risk of mortality among participants (36). Furthermore, smokers in our study may not have been exposed to smoking long enough for conditions to appear; we did not evaluate exposure time.

The lack of association between multimorbidity trajectories and physical activity can be explained by survival bias. A previous study of the *Como Vai?* sample described a higher risk of death among participants in the lowest tertile of overall physical activity compared with participants in the second and third tertiles of physical activity in 3 years of follow-up (13). Therefore, considering that the *Como Vai?* sample consisted of people who had already reached older age, that is, survived at least 6 decades, and people more exposed to risk factors are more likely to die prematurely, older adults with low levels of physical activity are more likely not to be monitored, reducing the magnitude of the associations found.

### Limitations

The study has several limitations. First, self-reported information on chronic conditions may be less accurate than objective measures or measures collected via medical records. Measures based on diagnostic criteria (eg, objective measurement of blood pressure and cholesterol) and symptoms (eg, depression, heart problems) are recommended for research on inequalities and multimorbidity but could not be used in this study because of restrictions in logistics and financing. Another limitation is that in the 6 years of study, we had information from only one-third of the sample at the second follow-up, because the study was halted as a result of the COVID-19 pandemic. On the other hand, the attrition analysis, which comprised participants with complete information (all 3 surveys), found consistency in the trajectories identified in the primary analysis.

### Conclusion

We found a high occurrence of chronic conditions in the study sample and identified 3 well-defined groups of older adults classified according to the number of diagnosed conditions that persisted over time. Approximately 1 in 5 adults aged 60 or older in Brazil lived with approximately 10 simultaneous chronic conditions during the study period. The increased risk of chronic conditions among older adults with no education or low levels of education poses a challenge to the health care of this population. The trajectories identified reflect phases of the study before the COVID-19 pandemic; future monitoring of this population may lead to different results related to the consequences of COVID-19.

## Figures and Tables

**Figure:**
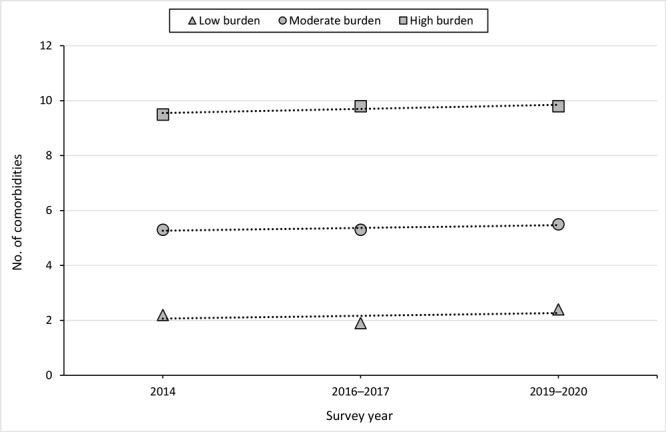
Results of an attrition analysis comparing the average number of chronic conditions, by year, across trajectories of disease burden among participants with information at all 3 time points in the *Como Vai*? study, Pelotas, Brazil. Of 440 study participants, 117 (26.6%) were categorized as having a low burden of chronic conditions, 254 (57.7%) moderate, and 69 (15.7%) high.

**Table:** 

Level	Mean (range) no. of chronic conditions, by year		
	2014	2016	2019
Low burden (n = 117)	2.2 (0–5)	1.9 (0–4)	2.4 (0–7)
Moderate burden (n = 254 )	5.3 (2–10)	5.3 (1–10)	5.5 (1–13)
High burden (n = 69)	9.5 (5–16)	9.8 (7–16)	9.8 (5–17)

## References

[1] Fortin M , Haggerty J , Almirall J , Bouhali T , Sasseville M , Lemieux M . Lifestyle factors and multimorbidity: a cross sectional study. *BMC Public Health.* 2014;14(1):686. 10.1186/1471-2458-14-686 24996220 PMC4096542

[2] Nunes BP , Batista SRR , Andrade FB , Souza PRB Jr , Lima-Costa MF , Facchini LA . Multimorbidity: the Brazilian Longitudinal Study of Aging (ELSI–Brazil). *Rev Saude Publica.* 2018;52(Suppl 2):10s. 10.11606/s1518-8787.2018052000637 30379288 PMC6254906

[3] Keomma K , Bousquat A , César CLG . Prevalence of multimorbidity in older adults in São Paulo, Brazil: a study with ISA–Capital. *Rev Saude Publica.* 2022;56:69. 10.11606/s1518-8787.2022056004252 35894406 PMC9337848

[4] Malta DC , Bernal RTI , Gomes CS , Cardoso LSM , Lima MG , Barros MBA . Inequalities in the use of health services by adults and elderly people with and without noncommunicable diseases in Brazil, 2019 National Health Survey. *Rev Bras Epidemiol.* 2021;24(Suppl 2):e210003. 10.1590/1980-549720210003.supl.2 34910057

[5] Miranda GMD , Mendes ACG , Silva ALA . Population aging in Brazil: current and future social challenges and consequences. *Rev Bras Geriatr Gerontol.* 2016;19(3):507–519. 10.1590/1809-98232016019.150140

[6] Oliveira-Figueiredo DST , Silva MPGPC , Feitosa PYO , Leite BC , Rocha FL , Andrade LDF . What is the burden of multimorbidity and the factors associated with its occurrence in elderly Brazilians? *Rev Bras Enferm.* 2024;77(1):e20220809. 10.1590/0034-7167-2022-0809 38716903 PMC11067935

[7] Afshar S , Roderick PJ , Kowal P , Dimitrov BD , Hill AG . Multimorbidity and the inequalities of global ageing: a cross-sectional study of 28 countries using the World Health Surveys. *BMC Public Health.* 2015;15(1):776. 10.1186/s12889-015-2008-7 26268536 PMC4534141

[8] Roberts KC , Rao DP , Bennett TL , Loukine L , Jayaraman GC . Prevalence and patterns of chronic disease multimorbidity and associated determinants in Canada. *Health Promot Chronic Dis Prev Can.* 2015;35(6):87–94. 10.24095/hpcdp.35.6.01 26302227 PMC4910465

[9] Delpino FM , Wendt A , Crespo PA , Blumenberg C , Teixeira DSDC , Batista SR , . Occurrence and inequalities by education in multimorbidity in Brazilian adults between 2013 and 2019: evidence from the National Health Survey. *Rev Bras Epidemiol.* 2021;24(suppl 2):e210016. 10.1590/1980-549720210016.supl.2 34910070

[10] Hsu HC . Trajectories of multimorbidity and impacts on successful aging. *Exp Gerontol.* 2015;66:32–38. 10.1016/j.exger.2015.04.005 25871727

[11] Jackson CA , Jones M , Tooth L , Mishra GD , Byles J , Dobson A . Multimorbidity patterns are differentially associated with functional ability and decline in a longitudinal cohort of older women. *Age Ageing.* 2015;44(5):810–816. 10.1093/ageing/afv095 26220988

[12] Quiñones AR , Liang J , Bennett JM , Xu X , Ye W . How does the trajectory of multimorbidity vary across Black, White, and Mexican Americans in middle and old age? *J Gerontol B Psychol Sci Soc Sci.* 2011;66(6):739–749. 10.1093/geronb/gbr106 21968384 PMC3198247

[13] Bielemann RM , LaCroix AZ , Bertoldi AD , Tomasi E , Demarco FF , Gonzalez MC , . Objectively measured physical activity reduces the risk of mortality among Brazilian older adults. *J Am Geriatr Soc.* 2020;68(1):137–146. 10.1111/jgs.16180 31592540

[14] Vargas PM , Schneider BC , Costa CS , César JA , Bertoldi AD , Tomasi E , . Age is the most important factor for change in body mass index and waist circumference in older people in southern Brazil. *Nutrition.* 2023;109:111956. 10.1016/j.nut.2022.111956 36863112

[15] Instituto Brasileiro de Geografia e Estatistica *. * *Censo Demografico 2010: Caracteristicas da Populacao e dos Domicilios. Resultados do Universo.* 2011. Accessed February 12, 2020. https://biblioteca.ibge.gov.br/visualizacao/periodicos/93/cd_2010_caracteristicas_populacao_domicilios.pdf

[16] GEOTER. Grupo de Pesquisa Geografia Política, Geopolítica e Territorialidades – Universidade Federal de Pelotas, 2020. Accessed May 8, 2023. https://ccs2.ufpel.edu.br/wp/2020/04/07/ufpel-realiza-mapeamento-da-populacao-de-risco-para-o-covid-19-no-espaco-urbano-de-pelotas

[17] Associação Brasileira de Empresas de Pesquisa. Critério de classificação econômica do Brasil. 2014. Accessed August 25, 2024. https://www.abep.org/criterio-brasil

[18] Ministério da Saúde. *Guia de Atividade Física para a População Brasileira.* 2021. Accessed September 20, 2023. http://bvsms.saude.gov.br/bvs/publicacoes/guia_atividade_fisica_populacao_brasileira.pdf

[19] Nagin D , Tremblay RE . Trajectories of boys’ physical aggression, opposition, and hyperactivity on the path to physically violent and nonviolent juvenile delinquency. *Child Dev.* 1999;70(5):1181–1196. 10.1111/1467-8624.00086 10546339

[20] Nagin DS . *Group-Based Modeling of Development.* Harvard University Press; 2005.

[21] Nagin DS , Odgers CL . Group-based trajectory modeling in clinical research. *Annu Rev Clin Psychol.* 2010;6(1):109–138. 10.1146/annurev.clinpsy.121208.131413 20192788

[22] Jones BL , Nagin DS . A note on a Stata plugin for estimating group-based trajectory models. *Sociol Methods Res.* 2013;42(4):608–613. 10.1177/0049124113503141

[23] Bähler C , Huber CA , Brüngger B , Reich O . Multimorbidity, health care utilization and costs in an elderly community-dwelling population: a claims data based observational study. *BMC Health Serv Res.* 2015;15(1):23. 10.1186/s12913-015-0698-2 25609174 PMC4307623

[24] Veras RP , Oliveira M . Envelhecer no Brasil: a construção de um modelo de cuidado [Aging in Brazil: the building of the healthcare model]. *Cien Saude Colet.* 2018;23(6):1929–1936. 10.1590/1413-81232018236.04722018 29972500

[25] World Health Organization. *UN Decade of Healthy Ageing: Plan of Action 2021–2030.* 2019. Accessed April 30, 2021. https://www.who.int/docs/default-source/decade-of-healthy-ageing/final-decade-proposal/decade-proposal-final-apr2020-en.pdf

[26] Nunes BP , Thumé E , Facchini LA . Multimorbidity in older adults: magnitude and challenges for the Brazilian health system. *BMC Public Health.* 2015;15(1):1172. 10.1186/s12889-015-2505-8 26602756 PMC4658761

[27] Agborsangaya CB , Majumdar SR , Sharma AM , Gregg EW , Padwal RS . Multimorbidity in a prospective cohort: prevalence and associations with weight loss and health status in severely obese patients. *Obesity (Silver Spring).* 2015;23(3):707–712. 10.1002/oby.21008 25682926

[28] Autenrieth CS , Kirchberger I , Heier M , Zimmermann AK , Peters A , Döring A , . Physical activity is inversely associated with multimorbidity in elderly men: results from the KORA-Age Augsburg Study. *Prev Med.* 2013;57(1):17–19. 10.1016/j.ypmed.2013.02.014 23485795

[29] Flores TR , Nunes BP , Assunção MCF , Bertoldi AD . Hábitos saudáveis: que tipo de orientação a população idosa está recebendo dos profissionais de saúde? [Healthy habits: what kind of guidance is the population receiving from health professionals?] *Rev Bras Epidemiol.* 2016;19(1):167–180. 10.1590/1980-5497201600010015 27167658

[30] Crimmins EM , Beltrán-Sánchez H . Mortality and morbidity trends: is there compression of morbidity? *J Gerontol B Psychol Sci Soc Sci.* 2011;66(1):75–86. 10.1093/geronb/gbq088 21135070 PMC3001754

[31] Woolf SH , Aron L , eds. *U.S. Health in International Perspective: Shorter Lives, Poorer Health.* National Academies Press; 2013. 24006554

[32] Cockerham WC , Hamby BW , Oates GR . The social determinants of chronic disease. *Am J Prev Med.* 2017;52(1S1):S5–S12. 10.1016/j.amepre.2016.09.010 27989293 PMC5328595

[33] Nagel G , Peter R , Braig S , Hermann S , Rohrmann S , Linseisen J . The impact of education on risk factors and the occurrence of multimorbidity in the EPIC-Heidelberg cohort. *BMC Public Health.* 2008;8(1):384. 10.1186/1471-2458-8-384 19014444 PMC2614432

[34] Kuo RN , Lai MS . The influence of socio-economic status and multimorbidity patterns on healthcare costs: a six-year follow-up under a universal healthcare system. *Int J Equity Health.* 2013;12(1):69. 10.1186/1475-9276-12-69 23962201 PMC3765221

[35] Benzeval M , Green MJ , Leyland AH . Do social inequalities in health widen or converge with age? Longitudinal evidence from three cohorts in the West of Scotland. *BMC Public Health.* 2011;11(1):947. 10.1186/1471-2458-11-947 22192620 PMC3265552

[36] Souza ACLG , Bortolotto CC , Bertoldi AD , Tomasi E , Demarco FF , Gonzalez MC , . All-cause mortality over a three-year period among community-dwelling older adults in Southern Brazil. *Rev Bras Epidemiol.* 2021;24:e210015. 10.1590/1980-549720210015 33825775

